# Sports vision training reduces digital eye strain in screen-exposed university students: a randomized controlled trial of visuomotor and visual-attentional adaptation

**DOI:** 10.3389/fnins.2026.1878323

**Published:** 2026-07-13

**Authors:** Huihui Zhong, Dongxu Gao

**Affiliations:** 1 Suzhou Polytechnic University, Suzhou, China; 2 Division of Sports Science and Physical Education, Tsinghua University, Beijing, China

**Keywords:** computer vision syndrome, digital eye strain, sensorimotor integration, sports vision training, stroboscopic visual training, visual fatigue, visuomotor coordination

## Abstract

**Background:**

Computer vision syndrome (CVS), or digital eye strain, is common among university students exposed to prolonged screen-based near work, yet most preventive approaches rely on passive strategies such as visual breaks, ergonomic advice, or optical filtering. This randomized controlled trial examined whether a 12-week sports vision training (SVT)-enhanced physical education program could reduce CVS symptoms in college students with high daily screen exposure.

**Methods:**

Two hundred undergraduate students were randomized to an SVT group or a control group, with 100 participants in each group and equal sex distribution. The SVT program integrated BACKNSHOU exercises, a structured sequence of eye, neck, shoulder, and back movements used for visual and postural preparation, Fitlight reaction tasks, SENAPTEC strobe-glasses training involving intermittent visual occlusion during visuomotor activities, multi-target tracking, and sport-based cognitive-motor activities. The control group continued standard physical education and received general 20-20-20 eye-use advice. CVS symptoms were assessed before and after intervention using CVS-SMART, covering visual-fatigue, ocular-surface, and neuromuscular/extraocular domains.

**Results:**

At baseline, 149 of 200 participants were classified as CVS-positive, corresponding to a prevalence of 74.5%, with no significant difference across sex-by-group strata. After 12 weeks, CVS-positive prevalence decreased from 76.0 to 44.0% in male SVT participants and from 72.0 to 38.0% in female SVT participants, whereas the control group showed only small, non-significant reductions. Self-reported symptom-specific analyses showed reductions in eye fatigue/soreness, difficulty focusing, red eyes, headache, and neck/shoulder/back pain in the SVT group. Generalized linear mixed models indicated lower post-intervention odds of symptom reporting across all five modeled symptoms, with group effects favoring SVT for all symptoms and reaching statistical significance for eye fatigue/soreness and red eyes.

**Conclusion:**

These findings suggest that dynamic, movement-based visual training may complement conventional CVS prevention strategies, although future studies incorporating objective ocular, oculomotor, and neurophysiological measures are needed to clarify mechanisms. This study extends sports vision training from athletic performance contexts to CVS symptom reduction in a non-athlete, screen-exposed student population.

## 1 Introduction

Computer vision syndrome (CVS), also known as digital eye strain, is a common visual health issue in populations that are increasingly using digital screens for long periods. CVS is more than just eye discomfort; it encompasses a spectrum of symptoms including eye fatigue, blurred vision, difficulty focusing, dry eye, redness, headache, and neck and shoulder discomfort (Jamaludin et al., 2025; Kahal et al., 2025). CVS is highly prevalent worldwide, with pooled estimates suggest that approximately two-thirds of digital device users experience CVS-related symptoms. The burden is especially significant for students in academic settings, who are now spending more time than ever in near-work activities, requiring them to maintain visual attention and continuously interact with laptops, phones, and other screen-based devices (Anbesu and Lema, 2023).

From a visual neuroscience perspective, CVS can be understood as a multidimensional condition involving ocular-surface stress, accommodative and vergence demand, oculomotor stability, attentional load, and extraocular musculoskeletal strain rather than a single ocular mechanism. Modern CVS assessment tools therefore include visual, ocular-surface, and extraocular symptom domains (Kaur et al., 2022; Pucker et al., 2024). Most CVS prevention and management strategies remain relatively passive, including scheduled visual breaks, artificial tears, blue-light filtering, and ergonomic education (Rahman and Sengupta, 2024). Although useful, these approaches do not directly train dynamic visual processing, visuomotor coordination, or adaptive visual attention (Pennartz et al., 2023; Nicolas et al., 2026).

Sports vision training (SVT) provides one such active approach. In athletic contexts, SVT is developed to enhance the visual and perceptual-motor skills which are essential when performing under dynamic conditions, such as visual search, peripheral vision, tracking, anticipation, reaction time, and hand–eye coordination (Appelbaum and Erickson, 2018). Stroboscopic visual training, a related SVT method, involves practicing tasks under intermittent visual input, thereby requiring individuals to use limited visual samples more efficiently and to rely more strongly on prediction, proprioception, and sensorimotor updating. Based on the literature on stroboscopic and sports vision training, these interventions may affect visual sensitivity (fovea), visual attention, visual-motor control, reaction time, and sport-related performance, though the evidence is diverse and the methodologies are variable (Wilkins and Appelbaum, 2020).

The rationale for applying SVT to CVS rests on experience-dependent plasticity in visual and visuomotor systems. Perceptual-learning and visual-rehabilitation studies indicate that adult visual performance can be modified through structured, task-specific training (Lu and Dosher, 2022). In screen-exposed students, shifting from static near-work to dynamic visual tracking, intermittent visual sampling, visuomotor responses, and sport-based attentional engagement may reduce perceived visual fatigue and related extraocular symptoms (Huxlin et al., 2009; Redondo et al., 2025).

However, despite its theoretical relevance, SVT has been rarely studied as a strategy for reducing CVS symptoms in non-athlete student populations. Much of the existing research on SVT has revolved around athletic performance, whereas CVS interventions have generally been based on ergonomic or optical approaches. This leaves an important translational gap between visual neuroscience, sport vision science and student visual-health promotion. A school-based SVT program incorporated into physical education could be particularly practical as it combines visual training with movement, postural regulation and structured activity and is feasible to implement in university settings.

The current randomized controlled trial examined if a 12-week SVT-enhanced physical education program could reduce CVS symptoms in college students with high daily screen time. The intervention included low-frequency visual masking, Fitlight reaction tasks, SENAPTEC strobe glasses, multi-target tracking, and sport-based cognitive-motor activities in a staged training progression. CVS-SMART was used to assess CVS symptoms before and after the intervention, including the domains of visual-fatigue, ocular-surface and neuromuscular/extraocular symptoms. The hypothesis was that students who received SVT-enhanced physical education would show larger reductions in the CVS-positive classification and prevalence of symptoms than students who received standard physical education, and that these effects would be similar for male and female participants.

## 2 Materials and methods

### 2.1 Study design

This study was a randomized controlled trial to evaluate whether a structured sports vision training intervention would help reduce symptoms of computer vision syndrome among college students who have high daily screen time. The trial was a 12-week sports vision training enhanced physical education versus standard physical education. Assessments were administered at baseline and immediately after the 12-week intervention period. A detailed institutional study protocol specifying the eligibility criteria, intervention procedures, outcome assessment, and statistical analysis plan was reviewed and approved by the Science and Technology Ethics Review Committee of Suzhou Vocational University of Technology before participant enrolment.

The study was performed in Suzhou Vocational University, undergraduate students of School of Electronic Information Engineering. The intervention was delivered in the regular physical education context, to test whether a visually demanding movement-based training could be delivered in a scalable educational context. The primary outcome was change in computer vision syndrome symptom status using the CVS-SMART Scale. Secondary analyses were performed to examine changes in specific visual, ocular-surface, and neuromuscular symptoms such as eye soreness, difficulty in focusing, red eyes, headache, and neck, shoulder, or back pain. This randomized controlled trial was conducted and reported in accordance with the Consolidated Standards of Reporting Trials (CONSORT) guidelines to ensure the transparent and comprehensive reporting of the study design, analysis, and interpretation of results. A completed CONSORT checklist is provided as Supplementary Table S1.

### 2.2 Ethical approval and consent to participate

The study protocol was reviewed and approved by the Science and Technology Ethics Review Committee of Suzhou Vocational University of Technology (IRB-1-2026-002; March 31, 2026). Prior to enrolment, all participants provided written informed consent. The study was conducted in compliance with the Declaration of Helsinki and the institutional requirements for studies involving human participants.

### 2.3 Participants

A total of 200 undergraduates participated. The sample consisted of 100 males and 100 females. Participants were eligible if they were 18–25 years of age, were enrolled as students in an undergraduate program, and were regularly exposed to digital screens on a daily basis. Students with a history of organic eye disease including glaucoma or cataract, previous ocular surgery, current vision therapy, use of medication known to affect visual function, or any medical or musculoskeletal condition that prevented participation in regular physical education activities were excluded.

**Figure 1:**
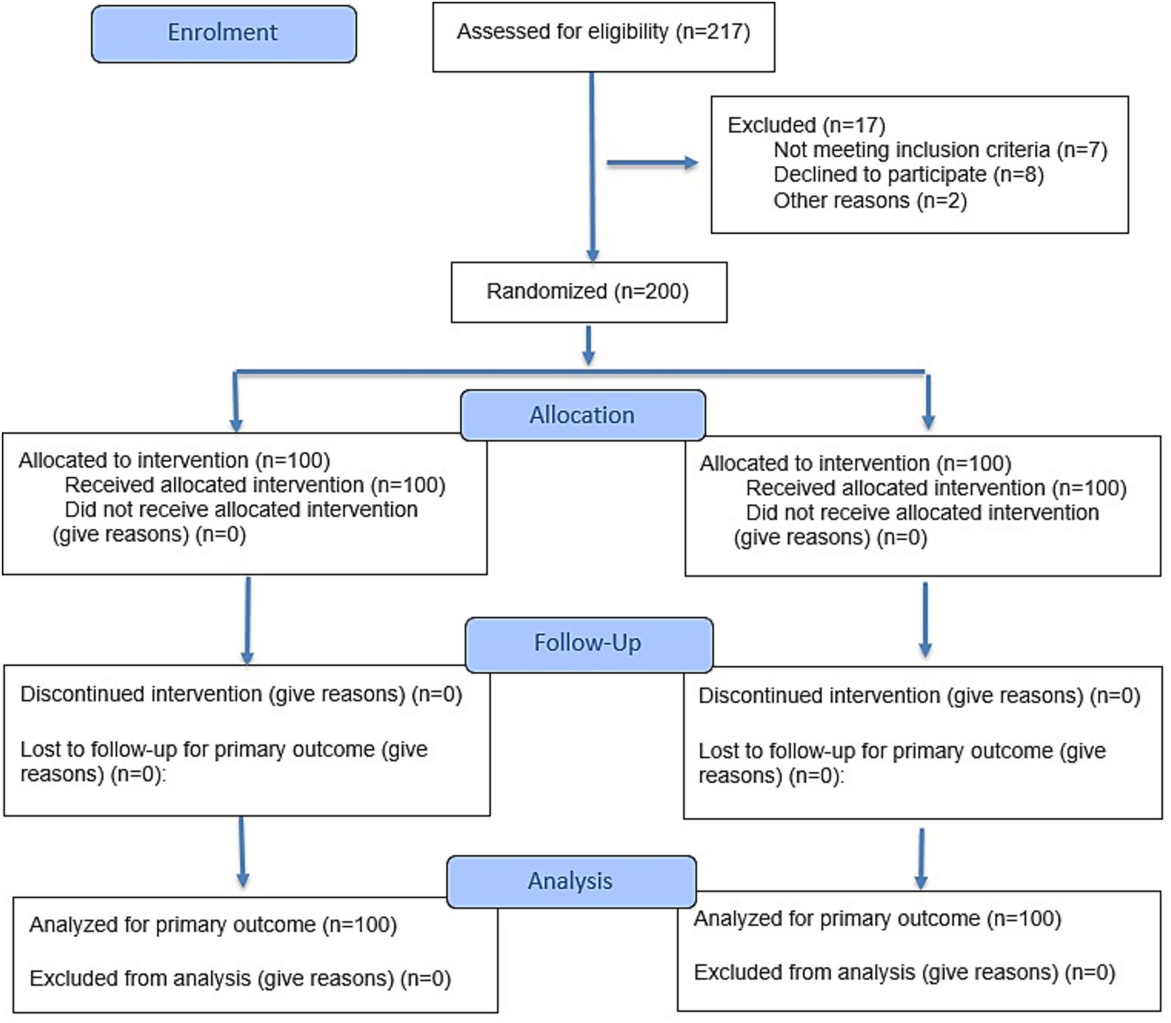
CONSORT flow diagram of participant recruitment, screening, randomization, allocation, follow-up, and analysis in the 12-week sports vision training trial.

Participants were randomly assigned to the sports vision training group or to the control group. In each group, there were 100 students with equal sex distribution: 50 male and 50 female participants in the intervention group and 50 male and 50 female participants in the control group. Randomization was carried out after baseline assessment using a computer-generated random number sequence in a 1:1 ratio. Group allocation was implemented after completion of the baseline CVS-SMART assessment. Because the intervention involved visible SVT equipment and instructor-supervised sport-based activities, participants and instructors could not be blinded to group allocation. Outcome assessment was based on self-administered CVS-SMART questionnaires, and no masked clinical assessor was involved. All randomized participants underwent the 12-week intervention and post-intervention assessment. No participants were lost to follow-up because assessments were integrated into scheduled university physical education sessions. The participant recruitment, randomization, allocation, follow-up, and analysis process is summarized in the CONSORT flow diagram (Figure 1).

Participants reported 6–8 h of daily screen exposure at baseline, primarily from laptops and smartphones. This high rate of screen use was pertinent to the study goal because students in electronic information engineering programs typically experience prolonged near-work and sustained demands on their digital vision.

### 2.4 Intervention

The participants in the intervention group took part in a 12-week sport vision training program that was incorporated into their physical education curriculum. Dynamic visual tasks were designed to be paired with sport-based motor activities in the intervention. The intervention was designed to promote controlled exposure to dynamic visual processing, visuomotor coordination, visual attention, peripheral awareness, reaction control and postural-neuromuscular regulation, rather than a passive eye-rest strategy.

The weekly sessions were 90 min long and contained three phases: preparation, core training and cool down.

The 15-min warm-up was intended to activate the visual and neuromuscular systems prior to visually demanding training. Participants performed a BACKNSHOU routine adapted from E-BACKNSHOU exercise, a range-of-motion and stretching approach targeting the eyes, neck, shoulders, back, and extremities in individuals with CVS-related extraocular complaints (Julianti et al., 2024). In the present study, the sequence consisted of eight-direction eye movements, joint mobilization, activation of upper body muscles and postural preparation drills. The activities were aimed at decreasing muscle tension, increasing proprioceptive readiness and preparing participants for the visual-motor tasks that followed.

The 60-min core training phase consisted of sport-related dynamic visual tasks. Participants engaged in Fitlight reaction training, which involved rapid detection and response to light stimuli while in motion. The exercises trained visual search, peripheral awareness, response selection and response speed. Participants also completed visual-occlusion tasks with SENAPTEC strobe glasses with masking frequencies set between 0.5 and 2 Hz depending on the stage of training. Sport activities such as basketball and table tennis were combined with strobe-based tasks to increase the dependence on anticipatory control, visuomotor updating and sensorimotor coordination. The exercises also included multi-target tracking, for example, dribbling while reacting to colored light signals or recognizing numbered visual targets while moving.

The cool-down period was 15 min long and consisted of visual relaxation exercises, followed by neck-resistance training with elastic bands. The resistance exercises consisted of three sets of push-and-pull movements for the neck, shoulder and upper-back muscles. This component was included as computer vision syndrome includes both ocular and extraocular symptoms and prolonged screen exposure is often related to neck, shoulder and back discomfort.

### 2.5 Training progression

The intervention was delivered in three stages over the 12-week period.

During the Foundation Stage, weeks 1–4, participants performed low frequency visual masking at 0.5 Hz and single light reaction tasks. The emphasis in this stage was on basic control of eye movement, accuracy of reaction, visual attention and simple visual-motor coordination.

During the Intensification Stage, weeks 5–8, difficulty of the visual tasks was increased. Participants were presented with medium-frequency visual masking at approximately 1 Hz, as well as multi-signal interference tasks. This stage involved more dynamic visual processing, attentional switching, visual search and response inhibition.

In the Practical Stage (weeks 9–12) participants engaged in more complex cognitive-motor tasks including multi-target tracking, sport specific movement and divided attention. The aim of this stage was to be as close to real life visual-motor requirements as possible.

Participants had to process several visual stimuli simultaneously while performing coordinated motor operations.

**Figure 2:**
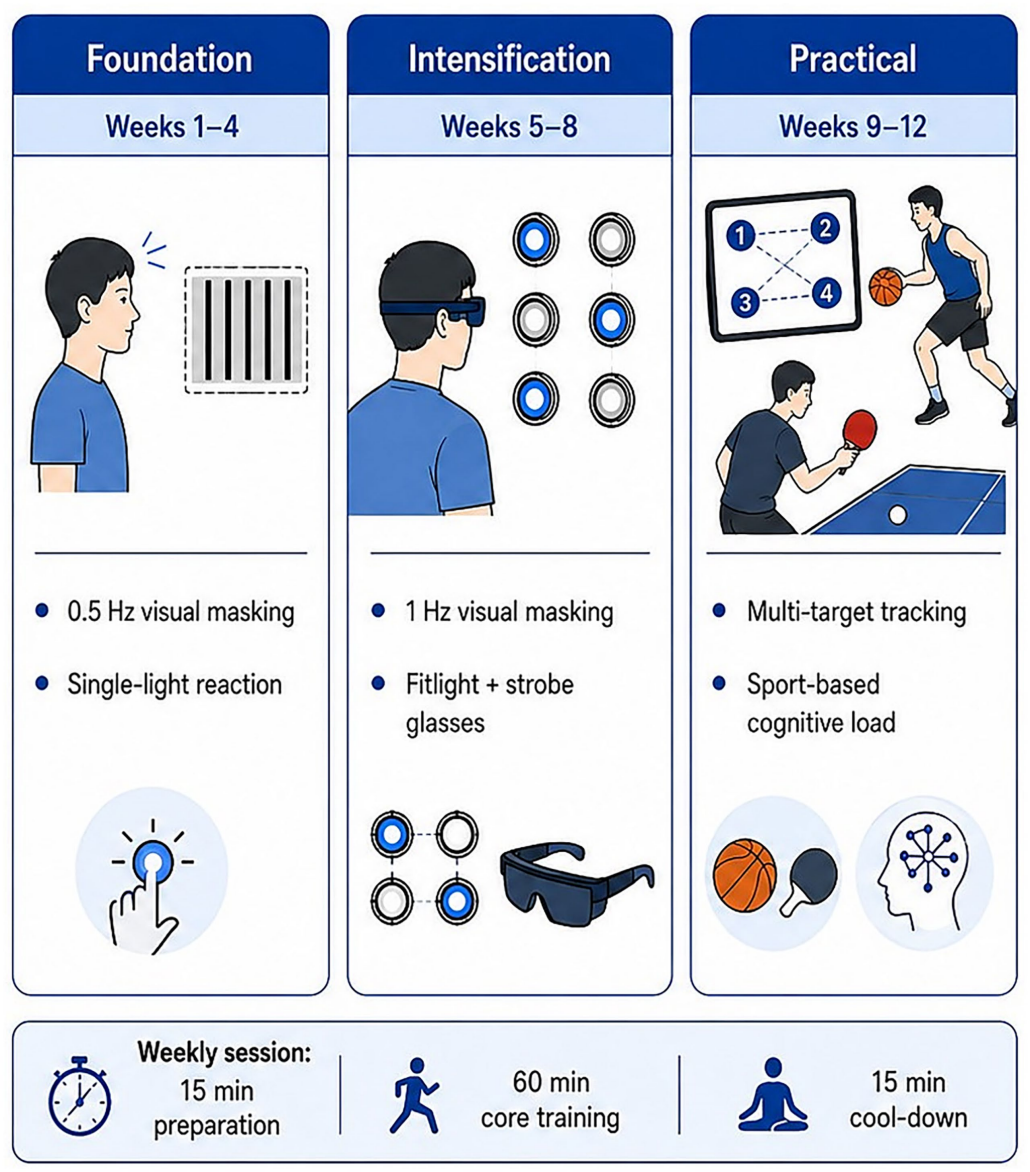
Staged progression of the 12-week sports vision training (SVT) intervention. During the Foundation stage (weeks 1–4), participants performed low-frequency visual masking (0.5 Hz) and single-light reaction tasks to develop basic visual attention and visuomotor control. During the Intensification stage (weeks 5–8), training incorporated Fitlight reaction exercises and stroboscopic visual training with approximately 1 Hz visual masking. During the Practical stage (weeks 9–12), participants engaged in multi-target tracking and sport-based cognitive-motor tasks designed to simulate dynamic real-world visual demands. Each weekly session included 15 min of preparation, 60 min of core training, and 15 min of cool-down exercises.

Participants in the intervention group were assigned daily after-school SVT tasks in addition to weekly class-based training. These tasks consisted of about 15 min of dynamic vision drills and 10 min of BACKNSHOU exercises. Compliance was monitored by Fitlight system records (e.g., reaction-time and error-rate outputs) and teacher supervision via a mobile app. In the intervention group, 85% of the students completed at least 80% of the tasks assigned to them. Figure 2 illustrates the staged progression of the 12-week sports vision training program, including the transition from foundational visual masking and simple reaction tasks to integrated multi-target visuomotor and sport-based cognitive training.

### 2.6 Control condition

The control group participated in the standard university physical education program. Their classes involved general physical activities without structured visual-training components, strobe-glasses training, Fitlight reaction tasks or multi-target tracking exercises.

Control participants were instructed to practice the 20-20-20 rule of looking at an object approximately 20 feet away for 20 s after every 20 min of screen time. Compliance with this recommendation, however, was not tracked systematically. Thus, the control condition was typical physical education with general advice about eye use rather than an actively supervised program of visual training.

### 2.7 Outcome assessment

CVS-SMART scale was used to measure symptoms of computer vision syndrome before and after the 12-week intervention. The instrument is made up of 27 items rated from 0 to 2 where 0 means no symptom, 1 means mild symptom and 2 means severe symptom. The items include three domains: visual fatigue, ocular-surface symptoms, and neuromuscular or extraocular symptoms.

The area of visual-fatigue included eye soreness, focusing difficulty, blurred vision, and low concentration. Ocular-surface domain symptoms included dry eye, red eye, foreign-body sensation, itching, burning, tearing, and increased secretions. The neuromuscular or extraocular category included headache, neck, shoulder and back pain, low mood, appetite change and finger or wrist discomfort.

Domain raw scores were standardized to a 0–1 scale. The average of the normalized domain scores was used to calculate the total CVS-SMART score. Total score of 7–10 was taken as a positive CVS case, 5–6 high probability CVS and 3–4 low probability CVS. We also performed binary analyses for each of the key symptoms (eye soreness, difficulty focusing, red eyes, headache, and neck, shoulder, or back pain) in addition to total CVS classification.

All symptom outcomes were self-reported. Objective grading of the ocular surface, tear film assessment, accommodative amplitude testing, blink rate measurement, eye tracking, electrophysiology or neuroimaging were not performed. Therefore, the results should be considered as changes in the perceived symptoms of CVS and not as direct evidence of the ocular physiological or neural changes.

### 2.8 Statistical analysis

Descriptive statistics were employed to summarize participant characteristics, baseline CVS-SMART classification, and symptom prevalence. Categorical variables were presented as frequencies and percentages. Baseline comparability between study groups was assessed using chi-square tests.

Descriptive analyses of symptom prevalence pre- and post-intervention were summarized by group and gender. First, Chi-square tests were run to examine changes in symptom proportions within groups. These descriptive subgroup comparisons were interpreted with caution as the same participants were assessed pre and post-intervention and were not considered the primary inferential analysis.

The main inferential analysis was based on generalized linear mixed models with a logit link function for repeated binary symptom outcomes. Separate models were fitted for each symptom outcome. Group, time and sex were included as fixed effects. The group variable was intervention vs. control condition, time was post-intervention vs. baseline assessment, and sex was female vs. male participants. Random intercepts at the participant level were included to account for repeated measurements within subjects.

Model results were expressed as regression coefficients (on the log-odds scale), standard errors, z values, *p* values, and 95% confidence intervals. Coefficients can also be exponentiated and reported as odds ratios with 95% confidence intervals for ease of interpretation. Statistical significance was defined as *p* < 0.05. It was interpreted that effects with confidence intervals that crossed the null value were not statistically significant, even if the *p* values were close to the conventional threshold.

Statistical analyses were performed using SPSS version 27.0.

## 3 Results

### 3.1 Participant characteristics and baseline CVS status

A total of 200 undergraduate students were enrolled and randomized to either the sports vision training (SVT) group or the control group. Each group included 100 participants, with equal sex distribution: 50 male and 50 female students in the SVT group and 50 male and 50 female students in the control group. All participants completed the 12-week study period and post-intervention assessment.

At baseline, CVS symptom burden was high across the cohort (Table 1). Overall, 149 of 200 participants were classified as CVS-positive, corresponding to a baseline prevalence of 74.5%. A further 28 participants (14.0%) were classified as high-probability CVS cases, and 23 participants (11.5%) were classified as low-probability CVS cases. CVS-positive prevalence was 78.0% in female controls, 72.0% in female SVT participants, 72.0% in male controls, and 76.0% in male SVT participants. There was no significant difference in baseline CVS diagnostic classification across the four sex-by-group strata, χ^2^ = 4.399, *p* = 0.623, indicating adequate baseline comparability.

**Table 1:** Baseline CVS-SMART diagnostic classification by group and sex.

CVS-SMART classification	Female control *n* (%)	Female SVT *n* (%)	Male control *n* (%)	Male SVT *n* (%)	Total *n* (%)	χ^2^	*p*-value
CVS-positive	39 (78.0)	36 (72.0)	36 (72.0)	38 (76.0)	149 (74.5)	4.399	0.623
Low-probability CVS	4 (8.0)	4 (8.0)	8 (16.0)	7 (14.0)	23 (11.5)		
High-probability CVS	7 (14.0)	10 (20.0)	6 (12.0)	5 (10.0)	28 (14.0)		
Total	50	50	50	50	200		

### 3.2 Baseline symptom profile

Baseline CVS-SMART responses showed that symptoms were frequent across visual-fatigue, ocular-surface, and neuromuscular domains (Table 2). Visual symptoms were common: blurred vision was reported by 65.5% of participants, eye fatigue or soreness by 78.5%, distraction or reduced concentration by 84.5%, and difficulty focusing by 78.0%. Ocular-surface symptoms were also highly prevalent, including dry eye in 76.0%, red eyes in 81.0%, foreign-body sensation in 76.0%, itching or burning in 76.0%, increased tearing in 80.0%, and increased secretions in 81.5% of participants. Neuromuscular and extraocular symptoms were similarly common, with headache reported by 76.5% and neck, shoulder, or back pain by 81.5% of participants.

**Table 2:** Baseline prevalence of selected CVS-SMART symptoms.

Symptom category	Symptom	Total *n* (%)	Baseline group comparison *p*-value
Visual fatigue	Blurred vision	131 (65.5)	0.770
	Eye fatigue/soreness	157 (78.5)	0.483
	Difficulty focusing	156 (78.0)	0.818
Visual attention	Distraction/reduced concentration	169 (84.5)	0.721
Ocular surface	Dry eye	152 (76.0)	0.883
	Red eyes	162 (81.0)	0.988
	Foreign-body sensation	152 (76.0)	0.778
	Itching or burning	152 (76.0)	0.974
	Increased tearing	160 (80.0)	0.969
	Increased secretions	163 (81.5)	0.947
Neuromuscular/extraocular	Headache	153 (76.5)	0.913
	Neck/shoulder/back pain	163 (81.5)	0.992

There were no statistically significant baseline differences across the four sex-by-group strata for the assessed symptom items, indicating that the SVT and control groups were comparable before the intervention.

### 3.3 Change in CVS diagnostic status after intervention

**Figure 3:**
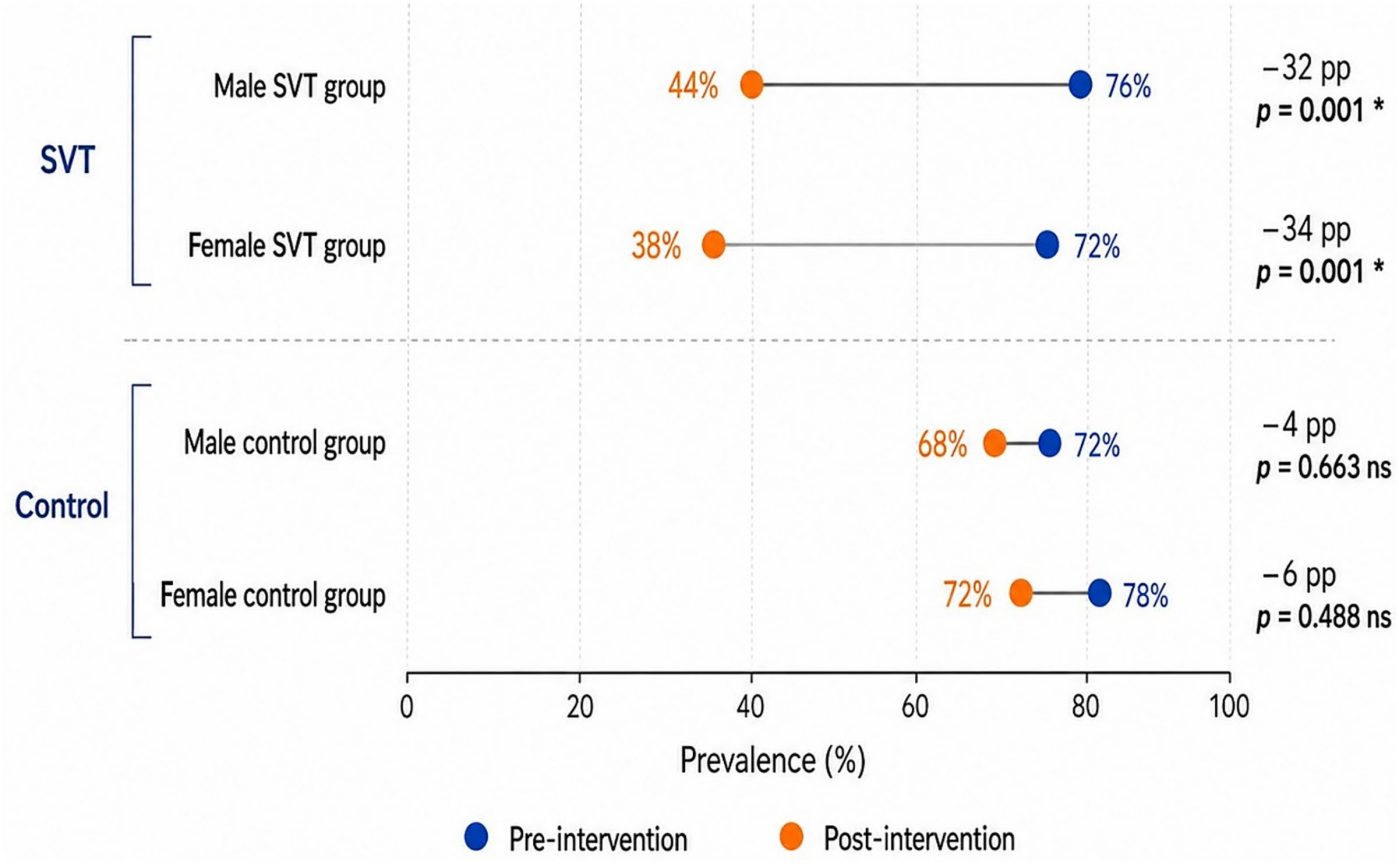
Pre- to post-intervention change in CVS-positive prevalence by group and sex. Slope plot showing the proportion of participants classified as CVS-positive before and after the 12-week intervention. Blue markers indicate pre-intervention prevalence and orange markers indicate post-intervention prevalence. CVS-positive prevalence decreased substantially in the SVT group but changed only slightly in the control group. CVS, computer vision syndrome; SVT, sports vision training.

After the 12-week intervention, CVS-positive classification decreased substantially in the SVT group but changed only slightly in the control group (Figure 3). Among male SVT participants, CVS-positive prevalence decreased from 76.0% before intervention to 44.0% after intervention, corresponding to a 32.0 percentage-point reduction, χ^2^ = 10.667, *p* = 0.001. Among female SVT participants, CVS-positive prevalence decreased from 72.0 to 38.0%, corresponding to a 34.0 percentage-point reduction, χ^2^ = 11.677, *p* = 0.001. In contrast, changes in the control group were small and not statistically significant. CVS-positive prevalence decreased from 72.0 to 68.0% in male controls, χ^2^ = 0.190, *p* = 0.663, and from 78.0 to 72.0% in female controls, χ^2^ = 0.480, *p* = 0.488.

### 3.4 Symptom-specific changes in the SVT group

The SVT group showed reductions across visual-fatigue, visual-attentional, ocular-surface, and neuromuscular symptom domains (Table 3). Red-eye symptoms decreased from 82.0 to 54.0% in male SVT participants and from 80.0 to 52.0% in female SVT participants, with both reductions reaching statistical significance.

**Table 3:** Symptom-specific changes in the SVT group.

Domain	Symptom	Male SVT pre *n* (%)	Male SVT post *n* (%)	*p*-value	Female SVT pre *n* (%)	Female SVT post *n* (%)	*p*-value	Combined absolute change
Ocular surface	Red eyes	41 (82.0)	27 (54.0)	0.003	40 (80.0)	26 (52.0)	0.003	−28.0 percentage points
Visual fatigue	Eye fatigue/soreness	37 (74.0)	25 (50.0)	0.013	41 (82.0)	23 (46.0)	<0.001	−30.0 percentage points
	Difficulty focusing	39 (78.0)	29 (58.0)	0.032	41 (82.0)	28 (56.0)	0.005	−23.0 percentage points
Visual attention	Distraction/reduced concentration	42 (84.0)	28 (56.0)	0.002	43 (86.0)	23 (46.0)	<0.001	−34.0 percentage points
Neuromuscular/extraocular	Headache	37 (74.0)	24 (48.0)	0.008	40 (80.0)	23 (46.0)	<0.001	−30.0 percentage points
	Neck/shoulder/back pain	41 (82.0)	27 (54.0)	0.003	41 (82.0)	26 (52.0)	0.001	−29.0 percentage points

Visual-fatigue symptoms also improved. Eye fatigue or soreness decreased from 74.0 to 50.0% in male SVT participants and from 82.0 to 46.0% in female SVT participants. Difficulty focusing decreased from 78.0 to 58.0% in male SVT participants and from 82.0 to 56.0% in female SVT participants. Distraction or reduced concentration decreased from 84.0 to 56.0% in male SVT participants and from 86.0 to 46.0% in female SVT participants.

Neuromuscular and extraocular symptoms followed the same pattern. Headache decreased from 74.0 to 48.0% in male SVT participants and from 80.0 to 46.0% in female SVT participants. Neck, shoulder, or back pain decreased from 82.0 to 54.0% in male SVT participants and from 82.0 to 52.0% in female SVT participants.

When male and female SVT participants were combined, the largest absolute reductions were observed for distraction or reduced concentration (−34.0 percentage points), CVS-positive classification (−33.0 percentage points), headache (−30.0 percentage points), neck/shoulder/back pain (−29.0 percentage points), eye fatigue/soreness (−30.0 percentage points), red eyes (−28.0 percentage points), and difficulty focusing (−23.0 percentage points).

### 3.5 Mixed-model analysis of key CVS symptoms

Generalized linear mixed models were fitted to evaluate predictors of binary symptom reporting while accounting for repeated pre- and post-intervention assessments. Across the five key symptom models, the time effect was consistently negative, indicating lower odds of symptom reporting after the intervention period. The post-intervention reduction was significant for eye fatigue/soreness, difficulty focusing, red eyes, headache, and neck/shoulder/back pain (Table 4).

**Table 4:** Generalized linear mixed-model estimates for key CVS symptom outcomes.

Symptom	Predictor	*β*	SE	z	*p*-value	95% CI for *β*
Eye fatigue/soreness	Group	−1.204	0.600	−2.007	0.045	−2.380 to −0.028
	Sex	−0.105	0.550	−0.191	0.849	−1.183 to 0.973
	Time	−1.609	0.580	−2.774	0.006	−2.746 to −0.472
Difficulty focusing	Group	−1.099	0.610	−1.802	0.072	−2.295 to 0.097
	Sex	−0.223	0.570	−0.391	0.696	−1.340 to 0.894
	Time	−1.386	0.590	−2.349	0.019	−2.542 to −0.230
Red eyes	Group	−1.204	0.590	−2.041	0.041	−2.360 to −0.048
	Sex	−0.105	0.560	−0.188	0.851	−1.203 to 0.993
	Time	−1.609	0.570	−2.823	0.005	−2.726 to −0.492
Headache	Group	−1.099	0.620	−1.773	0.076	−2.314 to 0.116
	Sex	−0.223	0.580	−0.384	0.701	−1.360 to 0.914
	Time	−1.386	0.600	−2.310	0.021	−2.562 to −0.210
Neck/shoulder/back pain	Group	−1.204	0.630	−1.911	0.056	−2.439 to 0.031
	Sex	−0.105	0.590	−0.178	0.859	−1.261 to 1.051
	Time	−1.609	0.610	−2.637	0.008	−2.805 to −0.413

*β*, log-odds coefficient; SE, standard error; CI, confidence interval. Group compares SVT with control; time compares post-intervention with baseline; sex compares female with male according to model coding.

**Figure 4:**
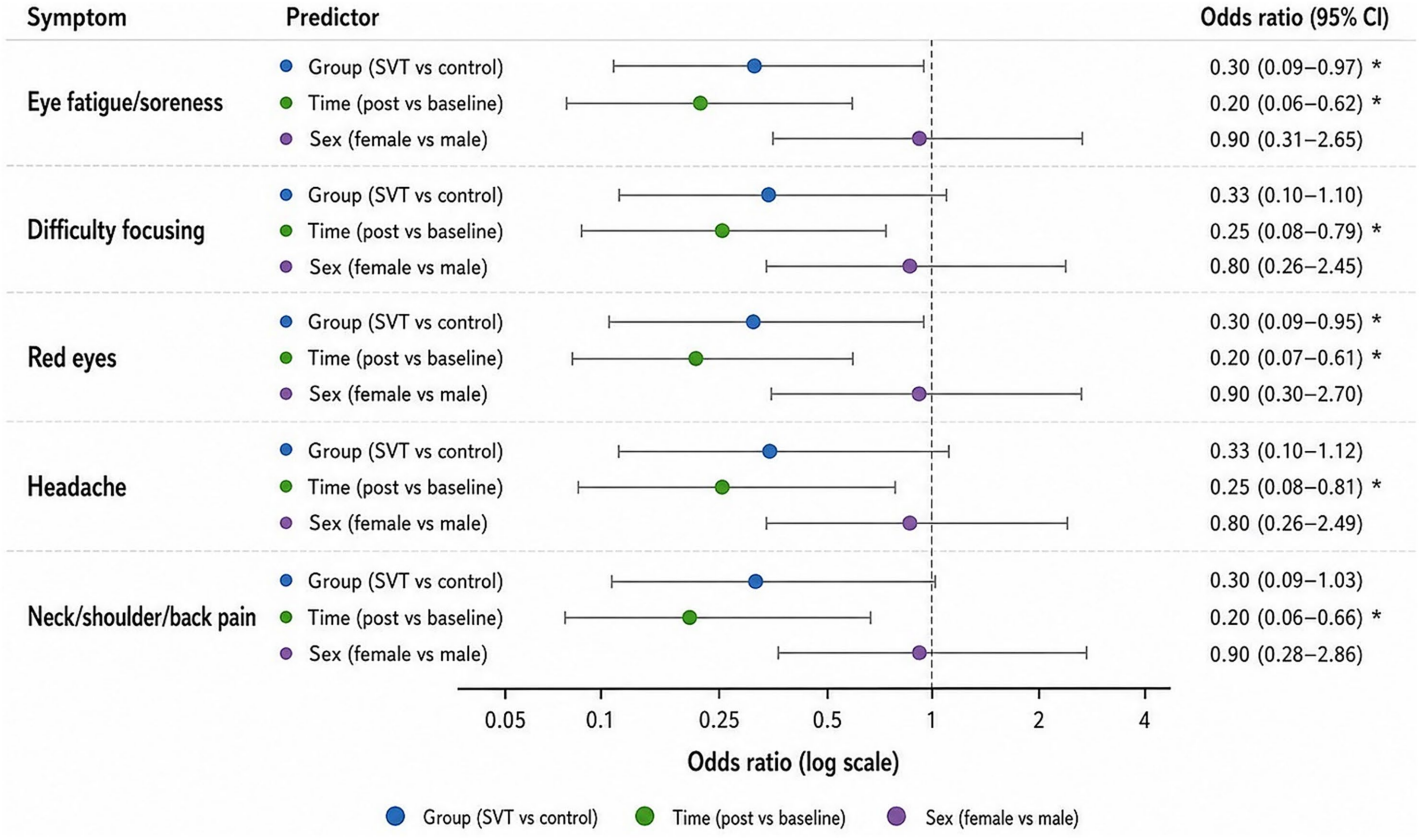
Forest plot of odds ratios and 95% confidence intervals from generalized linear mixed models for five CVS symptoms. Odds ratios below 1 indicate lower odds of symptom reporting. Time effects favored post-intervention improvement across all symptoms, group effects favored SVT for all symptoms, and sex showed no significant association. CVS, computer vision syndrome; SVT, sports vision training; CI, confidence interval.

The group effect was also negative across all models, indicating lower odds of symptom reporting in the SVT group than in the control group (Figure 4). This effect reached statistical significance for eye fatigue/soreness and red eyes. The group effects for difficulty focusing, headache, and neck/shoulder/back pain were in the same direction but did not reach the conventional *p* < 0.05 threshold. Sex was not significantly associated with symptom reporting in any model, indicating no evidence of a sex-dependent difference in the symptom response pattern.

Overall, the 12-week SVT intervention was associated with a significant reduction in CVS-positive classification and consistent reductions in self-reported visual-fatigue, ocular-surface and neuromuscular symptoms. The diagnostic reduction was significant for both male and female SVT participants, whereas the control group showed only minor non-significant changes. Mixed-model analyses corroborated descriptive findings and showed significant reductions in all five symptom outcomes modeled post-intervention and lower odds of several symptoms in the SVT group. Sex did not have significant effects, suggesting that the pattern of symptom reduction was largely similar for male and female participants.

## 4 Discussion

The study was a randomized controlled trial designed to evaluate the impact of a 12-week sports vision training (SVT)-based physical education program on the symptoms of computer vision syndrome (CVS) in college students with high daily screen time. The study yielded three principal results. First, the baseline symptom burden of CVS was high; 74.5% of participants were CVS-positive, and visual-fatigue, ocular-surface, and neuromuscular/extraocular symptoms were prevalent. Second, the SVT group had a significant decrease in CVS-positive classification (from 76.0 to 44.0% in males and from 72.0 to 38.0% in females), whereas the control group showed only small, non-significant reductions. Third, analyses at the symptom level showed widespread reductions in eye fatigue/soreness, difficulty focusing, red eyes, headache, and neck/shoulder/back pain, and mixed-model analyses showed lower post-intervention odds of reporting across all five modeled symptoms. Taken together, these findings suggest that a structured, movement-based visual training program may reduce perceived CVS symptom burden in students exposed to sustained digital visual demands.

The high baseline prevalence found in this study is in agreement with the broader epidemiological literature. A study reported a pooled prevalence of 66% of CVS worldwide and another meta-analysis reported a pooled prevalence of 69.0%, with substantial heterogeneity across countries, populations and assessment tools (Anbesu and Lema, 2023; Ccami-Bernal et al., 2024). Therefore, the 74.5% prevalence in the present cohort is not surprising especially as students reported approximately 6–8 h of daily screen exposure and were recruited from an electronic information engineering program. Prevalence among university participants was reported to be 73.7% in a recent university based study. Likewise, another study of technology students revealed a high burden of digital eye strain in screen-intensive academic settings (Almuqrashi et al., 2025; Likka et al., 2025). These comparisons indicate that the present cohort represents a high-risk but increasingly common student population.

The pattern of symptoms observed at baseline also agrees with recent work showing that CVS is multidimensional, not just ocular. Eye fatigue/soreness, difficulty in focusing, red eyes, headache and pain in neck/shoulder/back were all common in the present study. This pattern is consistent with extensive reviews that describe CVS as a syndrome with visual, ocular-surface and extraocular symptoms including blurred vision, dry eye, headache and musculoskeletal discomfort (Kaur et al., 2022). It has also been paralleled in study of working adults where digital eye strain was common and musculoskeletal symptoms such as neck, shoulder and back pain were reported by 94.3% of respondents using digital devices (Moore et al., 2024). Thus, the present findings support the notion that extended screen exposure imposes a dual visual-attentional and postural-neuromuscular load.

The magnitude of the improvement in CVS-positive classification following SVT is clinically significant. The prevalence of CVS-positivity was reduced by 32.0 percentage points in males and 34.0 percentage points in females in the SVT group, versus only 4.0 and 6.0 percentage points in the control group. This difference is important because both groups continued with physical education but only the SVT group received structured visual-motor training. The difference therefore suggests that the general physical education alone was insufficient to explain the observed improvement. Instead, the inclusion of dynamic visual tracking, intermittent visual occlusion, peripheral cue detection, reaction tasks, and multi-target sport-based training components may have helped to reduce the symptoms (Jothi et al., 2025).

This finding adds to the existing literature on CVS interventions. Previous interventions have focused on eye-rest strategies, ergonomic education, screen-use modification, or optical filtering. For example, the 20-20-20 rule is a common recommendation and has been reported to reduce some symptoms of digital eye strain and dry eye, but evidence suggests that short-term implementation may not be sufficient to improve binocular vision or objective dry-eye signs (Alghamdi and Alrasheed, 2020; Talens-Estarelles et al., 2023). Blue-light filtering lenses have also gained significant public attention, but a study concluded that blue-light filtering spectacles may not meaningfully reduce eye strain associated with computer use over short-term follow-up (Downie et al., 2019). Unlike these passive or exposure-reduction strategies, the proposed intervention actively trained visual-motor processing under dynamic conditions. This difference may be important as CVS may not be fully addressed by reducing exposure alone, but may also need interventions that improve tolerance to visual load, attentional switching, gaze variability and postural regulation.

The present findings are also relevant for research on eye exercises and combined eye–neck interventions. A study showed that eye exercises decreased the scores of CVS in medical students, which suggests that systematic ocular activity can decrease the symptom burden in students exposed to screens (Wang et al., 2023). Similarly, a recent study by Kannappan et al. investigated an exercise package for eye and neck strain and found that combined exercise may help with eye and neck symptoms, which is conceptually closer to the present intervention than purely optical or educational strategies (Kannappan et al., 2024). But unlike conventional eye exercises, the current SVT program puts visual tasks into a movement-rich sports context. Fitlight reaction tasks, SENAPTEC strobe-glasses training, and multi-target tracking likely require higher visual search, peripheral awareness, temporal sampling and visual-motor coordination demands compared to static eye exercises alone (Jothi et al., 2025). This could explain improvements at both the visual and neuromuscular levels.

From a visual neuroscience perspective, the findings align with the notion that visual fatigue during prolonged exposure to screens may involve persistent demands on accommodation, convergence, oculomotor stability, attentional control, and sensorimotor posture (Sigamani et al., 2022). Static near-work is characterized by relatively repetitive visual input and prolonged fixation, while SVT exposes participants to changing spatial cues, intermittent visual information, moving targets and coordinated body movement (Ren et al., 2025). Stroboscopic visual training is precisely focused on completing a task in conditions of intermittent vision, which encourages the use of limited visual samples, prediction, and sensorimotor updates (Das et al., 2023). Recently, a study described the stroboscopic visual training and its potential effects on visual attention and perceptual-motor performance, but also methodological challenges and the need for better-controlled studies (Wilkins and Appelbaum, 2020). In this context, the present trial aims to test the principles of stroboscopic and sports vision in a non-athlete population suffering from digital visual strain.

The reduction in eye fatigue/soreness and in difficulty focusing may be considered in terms of perceptual learning and adaptive visual processing. The adult visual system still has the capacity for experience-dependent plasticity, and research on perceptual learning has demonstrated that repeated, task-specific visual training can enhance visual performance. Huxlin et al. showed that complex visual motion processing can be relearnt after damage to V1 with specific training, and broader perceptual learning literature suggests that visual processing can be modified through practice (Huxlin et al., 2009; Sagi, 2011). The present study did not measure neural plasticity directly and therefore cannot claim cortical adaptation. The improvements in symptoms are however consistent with a model of behavioral adaptation where repeated dynamic visual tasks reduce the perceived strain of visual processing.

Recent sports vision literature also supports this importance of SVT for visual-motor performance. In a systematic review on sport vision, the development of visual skills was suggested to be useful for sports performance and injury prevention. While, another systematic review defined SVT as a training program designed to improve visual skills required for sport performance (Buscemi et al., 2024; Lochhead et al., 2024). More recent reviews on stroboscopic training found improvements in reaction time, decision making, hand–eye coordination and sport specific performance. However, the findings are heterogenous and dependent on training parameters and outcome measures (Luo et al., 2025; Wang et al., 2025). The present study differs from these athletic-performance studies by using SVT as a visual-health intervention rather than a performance-enhancement intervention. This translational shift is one of the main contributions of the study.

The reduction in red-eye symptoms deserves specific attention. Red eyes decreased from 82.0 to 54.0% in male SVT participants and from 80.0 to 52.0% in female SVT participants, and the GLMM group effect for red eyes favored the SVT group. Symptoms related to the ocular surface are frequently seen in CVS and are often associated with reduced blink rate, instability of the tear film, incomplete blinking and prolonged fixation when using a screen (Kaur et al., 2022). We did not objectively measure tear break-up time, blink rate, or ocular redness; thus the reduction in red-eye should be considered a self-reported improvement in symptoms rather than objective evidence of ocular-surface healing. One possible explanation is that SVT increased gaze variability and reduced the monotony of prolonged near fixation. Another explanation is that the supervised routine raised participants’ awareness of visual discomfort and encouraged healthier eye-use behavior outside of training. These hypotheses should be confirmed by objective ocular surface parameters.

The decrease in headache and neck/shoulder/back pain suggests that SVT may be affecting the extraocular component of CVS. This is important as several recent studies show a strong association of digital eye strain with musculoskeletal complaints. Moore et al. reported that UK working adults with digital eye strain experienced neck, shoulder, and back symptoms frequently (Moore et al., 2024). Similarly, Tanveer et al. reported common headache, eye strain, back discomfort, and neck/shoulder pain in people with CVS (Tanveer et al., 2025). The current SVT program consisted of BACKNSHOU exercises, sport-based movement, postural activation, and elastic-band neck/shoulder resistance. These elements may have reduced static postural load and upper-body muscular tension that may have contributed to the additional extraocular symptom improvements. This sets the intervention apart from eye-only approaches and lends support to a more comprehensive sensorimotor model of CVS.

Although the present study did not directly measure physiological change, several plausible pathways may explain how SVT could reduce CVS symptoms. First, dynamic gaze shifts, target tracking, and visual-reaction tasks may interrupt prolonged static near fixation and reduce sustained accommodative and vergence load. Second, intermittent visual occlusion and multi-target tracking may stimulate oculomotor stability, prediction, attentional switching, and visuomotor updating, which may improve tolerance to visually demanding tasks. Third, sport-based movement and BACKNSHOU/neck–shoulder exercises may reduce static cervical and upper-back muscle load associated with prolonged screen use. Fourth, increased gaze variability and supervised breaks from screen exposure may indirectly support blink behavior and ocular-surface comfort. These mechanisms remain hypothetical because tear-film parameters, accommodative function, blink rate, eye tracking, and neurophysiological outcomes were not collected in the present study.

A major implication of this study is that CVS prevention might benefit from a shift from passive visual hygiene to active visual-motor training. Educational reminders, visual breaks, ergonomic advice, and optical approaches may still be helpful but do not directly challenge dynamic visual attention, peripheral detection, reaction control, or sensorimotor coordination. The present intervention addressed these domains via a phased training design. This staged progression may be particularly suitable for university environments since it can be integrated into physical education without requiring a clinical setting.

From a practical implementation perspective, however, equipment-based SVT requires planning and institutional support. Fitlight systems and stroboscopic glasses may involve initial procurement costs, instructor training, device maintenance, and scheduling within existing physical education classes. Feasibility may therefore vary across institutions depending on available resources. A scalable approach could involve phased implementation, shared equipment sets, teacher-supervised group circuits, and the use of lower-cost dynamic visual-reaction drills where specialized devices are not available. Thus, while the present intervention appears feasible within a structured university physical education setting, broader implementation would require consideration of cost, staff training, class size, safety supervision, and equipment accessibility.

Several limitations should also be acknowledged. First, all outcomes were self-reported through CVS-SMART. Symptom perception is clinically relevant, but the absence of objective ocular, oculomotor, and neurophysiological measures limits mechanistic interpretation. The present findings should therefore be interpreted as changes in perceived CVS symptoms rather than direct evidence of ocular-surface healing, accommodative improvement, oculomotor adaptation, or neural change. Second, the study was conducted at a single institution and recruited students from electronic information engineering programs, who reported 6–8 h of daily screen exposure. Although this high-screen-exposure population was appropriate for evaluating CVS symptoms, the findings may not generalize to students from other disciplines or to populations with lower daily screen time. Third, participant and instructor blinding was not feasible because the intervention used visible SVT equipment and supervised sport-based activities, which may have introduced expectation or attention effects. Fourth, the control group received standard physical education and 20-20-20 advice; however, compliance with the advice was not systematically monitored. This may have created unequal supervision, adherence monitoring, and participant attention between groups, which could have influenced the observed between-group differences. Fifth, the multi-component design of the SVT precludes the isolation of which element—Fitlight training, stroboscopic masking, BACKNSHOU, multi-target tracking, or sport based movement—contributed most strongly to symptom improvement.

Future studies should use prospectively designed, multicenter randomized controlled trials with attention-matched active control groups. Multi-arm trials comparing SVT with conventional eye exercises, general physical activity, supervised 20-20-20 reminders, ergonomic education, and combined interventions would help determine whether SVT has specific benefits beyond movement, supervision, and visual-break effects. Future studies should also include objective ocular measures, such as tear break-up time, ocular redness grading, blink rate, and dry-eye biomarkers; oculomotor and binocular-vision measures, such as accommodative amplitude and facility, vergence function, near point of convergence, fixation stability, and eye-tracking indices; and neurophysiological measures, such as EEG or functional near-infrared spectroscopy, to examine visual attention and sensorimotor integration. Dose–response studies are also needed to determine optimal strobe frequency, session duration, training intensity, and maintenance schedules.

## 5 Conclusion

In conclusion, the present study provides randomized evidence that a structured SVT-enhanced physical education program can decrease self-reported CVS symptoms in college students with high screen exposure. The intervention was associated with significant decreases in CVS positive classification and broad improvements in visual-fatigue, ocular-surface and neuromuscular/extraocular symptom domains. Whereas passive approaches like filtering blue light or giving advice about visual breaks without supervision are used, SVT provides an active, movement-based approach that includes dynamic visual processing and sensorimotor coordination. However, because the present study used symptom-based outcomes, the findings should be interpreted as changes in behavior and self-reported symptoms rather than direct evidence of ocular physiological or neural change. Future studies using objective ocular and neurophysiological measures are required to determine the mechanisms and sustainability of SVT effects in CVS prevention and management.

## Data Availability

The raw data supporting the conclusions of this article will be made available by the authors, without undue reservation.
