# Supplementary material for: Sports vision training reduces digital eye strain in screen-exposed university students: a randomized controlled trial of visuomotor and visual-attentional adaptation
Source: Front Neurosci. 2026 Jul 13;20:1878323. doi: 10.3389/fnins.2026.1878323 (PMC13403497; doi:10.3389/fnins.2026.1878323)
Supplement: Supplementary file 1 [file Table_1.docx]

**Supplementary Table S1. CONSORT 2025 checklist of information to include when reporting a randomised trial***

| **Section / Topic** | **No** | **CONSORT 2025 checklist item description** | **Where reported (section/table/figure)** |
| --- | --- | --- | --- |
| **Title and abstract** | | |  |
| Title and structured abstract | 1a | Identification as a randomised trial | Article title |
|  | 1b | Structured summary of the trial design, methods, results, and conclusions | Abstract |
| **Open science** | | |  |
| Trial registration | 2 | Name of trial registry, identifying number (with URL) and date of registration | Not reported |
| Protocol and statistical analysis plan | 3 | Where the trial protocol and statistical analysis plan can be accessed | Materials and methods - Study design |
| Data sharing | 4 | Where and how the individual de-identified participant data (including data dictionary), statistical code and any other materials can be accessed | Data availability statement |
| Funding and conflicts of interest | 5a | Sources of funding and other support (e.g., supply of drugs), and role of funders in the design, conduct, analysis and reporting of the trial | Funding |
|  | 5b | Financial and other conflicts of interest of the manuscript authors | Conflict of interest |
| **Introduction** | | |  |
| Background and rationale | 6 | Scientific background and rationale | Introduction |
| Objectives | 7 | Specific objectives related to benefits and harms | Introduction (final paragraph) |
| **Methods** | | |  |
| Patient and public involvement | 8 | Details of patient or public involvement in the design, conduct and reporting of the trial | Not applicable |
| Trial design | 9 | Description of trial design including type of trial (e.g., parallel group, crossover), allocation ratio, and framework (e.g., superiority, equivalence, non-inferiority, exploratory) | Materials and methods - Study design; Participants |
| Changes to trial protocol | 10 | Important changes to the trial after it commenced including any outcomes or analyses that were not prespecified, with reason | Not applicable |
| Trial setting | 11 | Settings (e.g., community, hospital) and locations (e.g., countries, sites) where the trial was conducted | Materials and methods - Study design |
| Eligibility criteria | 12a | Eligibility criteria for participants | Materials and methods - Participants |
|  | 12b | If applicable, eligibility criteria for sites and for individuals delivering the interventions (e.g., surgeons, physiotherapists) | Not applicable |
| Intervention and comparator | 13 | Intervention and comparator with sufficient details to allow replication. If relevant, where additional materials describing the intervention and comparator (e.g., intervention manual) can be accessed | Materials and methods - Intervention; Training progression; Control condition; Figure 2 |
| Outcomes | 14 | Pre-specified primary and secondary outcomes, including the specific measurement variable (e.g., systolic blood pressure), analysis metric (e.g., change from baseline, final value, time to event), method of aggregation (e.g., median, proportion), and time point for each outcome | Materials and methods - Study design; Outcome assessment |
| Harms | 15 | How harms were defined and assessed (e.g., systematically, non-systematically) | Not applicable |
| Sample size | 16a | How sample size was determined, including all assumptions supporting the sample size calculation | Not reported |
|  | 16b | Explanation of any interim analyses and stopping guidelines | Not applicable |
| **Randomisation:** |  |  |  |
| Sequence generation | 17a | Who generated the random allocation sequence and the method used | Materials and methods - Participants |
|  | 17b | Type of randomisation and details of any restriction (e.g., stratification, blocking and block size) | Materials and methods - Participants |
| Allocation concealment mechanism | 18 | Mechanism used to implement the random allocation sequence (e.g., central computer/telephone; sequentially numbered, opaque, sealed containers), describing any steps to conceal the sequence until interventions were assigned | Materials and methods - Participants |
| Implementation | 19 | Whether the personnel who enrolled and those who assigned participants to the interventions had access to the random allocation sequence | Not reported |
| Blinding | 20a | Who was blinded after assignment to interventions (e.g., participants, care providers, outcome assessors, data analysts) | Materials and methods - Participants |
|  | 20b | If blinded, how blinding was achieved and description of the similarity of interventions | Materials and methods - Participants (not blinded) |
| Statistical methods | 21a | Statistical methods used to compare groups for primary and secondary outcomes, including harms | Materials and methods - Statistical analysis |
|  | 21b | Definition of who is included in each analysis (e.g., all randomised participants), and in which group | Materials and methods - Participants; Statistical analysis; Results - Participant characteristics and baseline CVS status |
|  | 21c | How missing data were handled in the analysis | Materials and methods - Participants; Results - Participant characteristics and baseline CVS status (no losses) |
|  | 21d | Methods for any additional analyses (e.g., subgroup and sensitivity analyses), distinguishing prespecified from post-hoc | Materials and methods - Statistical analysis |
| **Results** | | |  |
| Participant flow, including flow diagram | 22a | For each group, the numbers of participants who were randomly assigned, received intended intervention, and were analysed for the primary outcome | Materials and methods - Participants; Results - Participant characteristics and baseline CVS status; Figure 1 |
|  | 22b | For each group, losses and exclusions after randomisation, together with reasons | Materials and methods - Participants; Results - Participant characteristics and baseline CVS status; Figure 1 |
| Recruitment | 23a | Dates defining the periods of recruitment and follow-up for outcomes of benefits and harms | Not reported |
|  | 23b | If relevant, why the trial ended or was stopped | Results - Participant characteristics and baseline CVS status; Figure 1 (trial completed) |
| Intervention and comparator delivery | 24a | Intervention and comparator as they were actually administered (e.g., where appropriate, who delivered the intervention/comparator, how participants adhered, whether they were delivered as intended [fidelity]) | Materials and methods - Intervention; Training progression; Control condition |
|  | 24b | Concomitant care received during the trial for each group | Not applicable |
| Baseline data | 25 | A table showing baseline demographic and clinical characteristics for each group | Results - Participant characteristics and baseline CVS status; Baseline symptom profile; Tables 1-2 |
| Numbers analysed,  outcomes and estimation | 26 | For each primary and secondary outcome, by group:  the number of participants included in the analysis  the number of participants with available data at the outcome time point result for each group, and the estimated effect size and its precision (such as 95% confidence interval)  for binary outcomes, presentation of both absolute and relative effect size | Results - Change in CVS diagnostic status; Symptom-specific changes; Mixed-model analysis; Tables 3-4; Figures 3-4 |
| Harms | 27 | All harms or unintended events in each group | Not applicable |
| Ancillary analyses | 28 | Any other analyses performed, including subgroup and sensitivity analyses, distinguishing pre-specified from post-hoc | Results - Symptom-specific changes; Mixed-model analysis; Tables 3-4; Figure 4 |
| **Discussion** | | |  |
| Interpretation | 29 | Interpretation consistent with results, balancing benefits and harms, and considering other relevant evidence | Discussion; Conclusion |
| Limitations | 30 | Trial limitations, addressing sources of potential bias, imprecision, generalisability, and, if relevant, multiplicity of analyses | Discussion (limitations paragraph) |

*We strongly recommend reading this statement in conjunction with the CONSORT 2025 Explanation and Elaboration and/or the CONSORT 2025 Expanded Checklist for important clarifications on all the items. We also recommend reading relevant CONSORT extensions. See [www.consort-spirit.org](http://www.consort-spirit.org).

Citation: Hopewell S, Chan AW, Collins GS, Hróbjartsson A, Moher D, Schulz KF, et al. CONSORT 2025 Statement: updated guideline for reporting randomised trials. BMJ. 2025; 388:e081123. <https://dx.doi.org/10.1136/bmj-2024-081123>.

© 2025 Hopewell et al. This is an Open Access article distributed under the terms of the Creative Commons Attribution License (<https://creativecommons.org/licenses/by/4.0/>), which permits unrestricted use, distribution, and reproduction in any medium, provided the original work is properly cited.
